# Association between plasma metabolites and gene expression profiles in five porcine endocrine tissues

**DOI:** 10.1186/1297-9686-43-28

**Published:** 2011-07-25

**Authors:** Bin Yang, Anna Bassols, Yolanda Saco, Miguel Pérez-Enciso

**Affiliations:** 1 Department of Food and Animal Science, Veterinary School, Universitat Autònoma de Barcelona, Bellaterra, 08193 Spain; 2 Centre for Research in Agricultural Genomics (CRAG), Bellaterra, 08193 Spain; 3 Key Laboratory for Animal Biotechnology of Jiangxi Province and the Ministry of Agriculture of China, Jiangxi Agricultural University, Nanchang, 330045, China; 4 Department of Biochemistry and Molecular Biology, Veterinary School, Universitat Autònoma de Barcelona, Bellaterra, 08193 Spain; 5 ICREA, Passeig Lluís Companys, 23; 08010 Barcelona, Spain

## Abstract

**Background:**

Endocrine tissues play a fundamental role in maintaining homeostasis of plasma metabolites such as non-esterified fatty acids and glucose, the levels of which reflect the energy balance or the health status of animals. However, the relationship between the transcriptome of endocrine tissues and plasma metabolites has been poorly studied.

**Methods:**

We determined the blood levels of 12 plasma metabolites in 27 pigs belonging to five breeds, each breed consisting of both females and males. The transcriptome of five endocrine tissues i.e. hypothalamus, adenohypophysis, thyroid gland, gonads and backfat tissues from 16 out of the 27 pigs was also determined. Sex and breed effects on the 12 plasma metabolites were investigated and associations between genes expressed in the five endocrine tissues and the 12 plasma metabolites measured were analyzed. A probeset was defined as a quantitative trait transcript (QTT) when its association with a particular metabolic trait achieved a nominal P value < 0.01.

**Results:**

A larger than expected number of QTT was found for non-esterified fatty acids and alanine aminotransferase in at least two tissues. The associations were highly tissue-specific. The QTT within the tissues were divided into co-expression network modules enriched for genes in Kyoto Encyclopedia of Genes and Genomes or gene ontology categories that are related to the physiological functions of the corresponding tissues. We also explored a multi-tissue co-expression network using QTT for non-esterified fatty acids from the five tissues and found that a module, enriched in hypothalamus QTT, was positioned at the centre of the entire multi-tissue network.

**Conclusions:**

These results emphasize the relationships between endocrine tissues and plasma metabolites in terms of gene expression. Highly tissue-specific association patterns suggest that candidate genes or gene pathways should be investigated in the context of specific tissues.

## Background

In recent years, high-throughput genomic technologies have accelerated the discovery of new causal mutations and made the study of biological systems more accessible than ever. This is true not only in humans and model organisms but also in agriculturally important species like the pig. One major interest in the study of livestock species is that the strong selection pressure applied in breeding programs has resulted in breeds that are phenotypically extreme for many traits. In addition, such selection has indirectly acted on the transcriptome and the metabolome, but the resulting effects are much less studied, not to say understood, than those on external phenotypes like growth or fat deposition.

In humans and other animal species, the blood levels of molecules related to lipid, glucose and protein metabolism, such as non-esterified fatty acids, triglyceride, glucose and alanine aminotransferase (ALT), reflect nutritional and disease status. In livestock species, the abundance of plasma metabolites can be associated with agriculturally important traits like growth and fatness [1]. Among the major livestock species, pig is a good model for human diseases such as atherosclerosis [2]. Genetic mapping studies have identified several genetic loci affecting blood metabolites in both human and pig populations [3,4]. Ideally, the functions of genes need to be defined in the context of relevant tissues and gene expression networks. Most of the studies that combine gene expression network and data on plasma metabolites have been primarily carried out on liver and adipose tissues [5,6]. However, endocrine glands, by secreting hormones, also play a pivotal role in maintaining the homeostasis of plasma metabolites, either directly or indirectly. Despite the importance of these tissues, the relationship between endocrine transcriptome and plasma metabolites is not well known. In addition, most existing analyses have considered tissues separately although complex traits like obesity or metabolite blood levels involve molecular networks both within and between multiple tissues.

In the work reported here, we have analyzed the association between the transcriptome of five endocrine tissues (hypothalamus, adenohypophysis, thyroid gland, gonad and fat tissue) and 12 plasma metabolites in pig. Since the study was carried out on pigs belonging to different breeds but managed and sacrificed simultaneously, we could also investigate the existence of any genetic (breed) effect on the metabolites analyzed. The plasma metabolites studied here play a fundamental role in the basal metabolism (glucose, cholesterol, triglyceride and non-esterified fatty acids, alanine aminotransferase), or the inflammatory response (haptoglobin, pig major acute phase protein). The term "quantitative trait transcript" or QTT refers to a probeset, the expression of which is significantly associated (P < 0.01) with a particular metabolic trait. Gene co-expression networks, were inferred both for each tissue separately and for all tissues together. We conclude that using a multi-tissue network provides key relevant information to understand the underlying regulation of the metabolites studied.

## Methods

### Animals and sample collection

Animal management and tissue collection procedures have been detailed elsewhere [7]. Briefly, 27 pigs from five breeds, Large White (N = 6), Landrace (N = 5), Duroc (N = 5), a Sino-European hybrid line (N = 5) and Iberian (N = 6), were bought from three breeding companies after weaning. All pigs were housed together in the university experimental farms and fed the same diet for two months. At 80 to 89 days of age and after 24 hours fasting, pigs were euthanized and sacrificed for blood and tissue sampling. All procedures were approved by the Ethical and Animal Welfare committee of the Universitat Autònoma de Barcelona (Spain).

### Phenotype measurements

Twelve plasma metabolites were measured in the 27 pigs. Briefly, after collecting and coagulating blood samples at room temperature, serum was separated from clots by centrifugation at 3000 rpm at 4°C for 20 min and stored at -80°C until use. Plasma metabolite concentrations were measured with the following methods: hexokinase assay for glucose, Ranbut assay (Randox Laboratories Ltd., UK) for 3-hydroxybutyrate, NEFA-C reagent (Wako Chemicals GmbH, Germany) for non-esterified fatty acids (NEFA), CHOD-PAP-method for cholesterol, immuno-inhibition method for high density lipoprotein cholesterol (HDL-C), selective protection method for low density lipoprotein cholesterol (LDL-C), GPO-PAP method for triglyceride, Biuret method for total protein and, methods recommended by IFCC (International Federation of Clinical Chemistry) for alanine aminotransferase (ALT) and alkaline phosphatase (ALP). Haptoglobin was assayed with the Phase Haptoglobin kit (colorimetric assay based on binding of haptoglobin to hemoglobin, Tridelta Ltd, Ireland) and pig major acute phase protein (PigMAP) levels with an ELISA kit (PigCHAMP ProEuropa, Segovia, Spain). All the assays were performed with an Olympus AU400 analyzer according to the manufacturer's recommendations.

### Microarray data

We used the GeneChip^® ^Porcine Genome Array from Affymetrix (Santa Clara CA) to profile the transcriptome of five endocrine tissues: hypothalamus (HYPO), adenohypophysis (AHYP), thyroid gland (THYG), gonads (GONA) from both male and female pigs, and backfat tissue (FATB) in 16 (four Large White, four Duroc, four Iberian and four from the Sino-European hybrid line) of the 27 pigs. Each breed consisted of two males and two females, except for the hybrid line with three males and one female [7]. Total RNA was extracted from 100 mg of tissue and RNA samples were cleaned, quantified, and adjusted to 500-1000 ng/μl. Five μg of total RNA were used to synthesize cDNA. Then, the 80 microarrays corresponding to 16 animals × five tissues were hybridized and scanned to generate signal intensities which were converted to CEL files by the GeneChip Operating Software (GCOS). All CEL files were adjusted for background noise and normalized using the GCRMA procedure [8] and the data was then used for subsequent analysis. The transcriptome data are deposited in the Gene Expression Omnibus (GEO) database under accession number [GEO:GSE14739].

### Data processing and analysis

We used a general linear regression model to investigate the effect of sex and breed on the biochemical traits:

where **y **is a vector of the studied metabolite measures.

The model applied to assess the strength of the association between metabolic traits and probesets was:

where probeset *i *is defined as a quantitative trait transcript (QTT) if its association with a particular biochemical trait achieves a nominal P value < 0.01. Since both breed and sex were adjusted in the regression analysis, the detected QTT for a particular metabolite represent general transcriptional effects in both breed and sex. The analysis were implemented using the GLM function in R [9]. The False Discovery Rates (FDR) of the associations were determined by permuting the labels of the phenotypes for 20 iterations, while preserving the correlation structure of the transcriptome.

#### Gene set enrichment analysis

A gene set enrichment analysis (GSEA) was implemented using R scripts downloaded from http://www.broadinstitute.org/gsea/ with a few modifications. In this analysis, the average value across probesets was used as the expression value of that gene in each individual when a gene was represented by more than one probeset. This reduced the 24,123 probesets to 18,017 unique genes. For each metabolic trait, we ranked the 18,017 genes according to their partial correlations with the metabolic trait under study (conditional on sex and breed). Then, an enrichment score measuring the extent to which a predefined set of genes (e.g., genes in a specific KEGG for Kyoto Encyclopedia of Genes and Genomes category) clustered at the top or the bottom of the ranks is calculated for each gene set. The normalized enrichment scores were used to measure the strength of the association between gene sets and the metabolic trait. The significance and FDR of the associations were determined by 1000 permutations [10].

#### Weighted gene co-expression network analysis

The gene expression data were corrected for sex and breed effects, and corresponding residuals were used to build up a weighted gene co-expression network using R package weighted gene co-expression network analysis (WGCNA) [11,12]. Briefly, a Pearson correlation matrix was first obtained and then transformed into an adjacency matrix A using a power function *a_ij _*= |*r_ij_*|*^β^*, where |*r_ij_*| is the absolute value of Pearson correlation coefficients between probeset *i *and probeset *j*, *a_ij _*is the element in A. The network connectivity (*K*) of probeset *i *is defined as  where index *j *corresponds to all probesets other than probeset *i *in the network, *N *is the overall number of transcripts studied [12]. The parameter *β *is chosen so that the connectivity distribution approximates a scale-free criterion, *P*(*K*) = *K^-r^*. The adjacency matrix was further transformed into a distance matrix through topological overlap-based dissimilarity measures; finally a dynamic clustering procedure was applied on the distance matrix to divide the entire co-expression network into multiple modules [12]. Similarly, the intramodular connectivity probeset *i *was defined as , where index *j *indicates all probesets other than probeset *i *in a specific module of size *N_m_*.

We also introduced a standardized inter-tissue connectivity of probeset *i*: , which measures the connection strength for a probeset *i *to probesets in external tissues, here index *l *indicates all the *N_ot _*probesets in tissues other than the tissue to which probeset *i *corresponds. The strength of connection between a pair of tissues with regard to gene expression is defined as , where *i *and *j *correspond to probesets in tissue 1 and tissue 2, and *N_1 _*and *N_2 _*are the number of probesets in tissue 1 and tissue 2, respectively.

#### Gene ontology (GO) and KEGG pathway enrichment analysis

The porcine Affymetrix probeset identifiers were converted into their human orthologs using the latest annotation file version (2010) from [13]. The gene category enrichment analyses were conducted using the Database for Annotation, Visualization and Integrated Discovery (DAVID) web-accessible program [14].

## Results

### Breed and sex differences for metabolite traits

**Figure 1:**
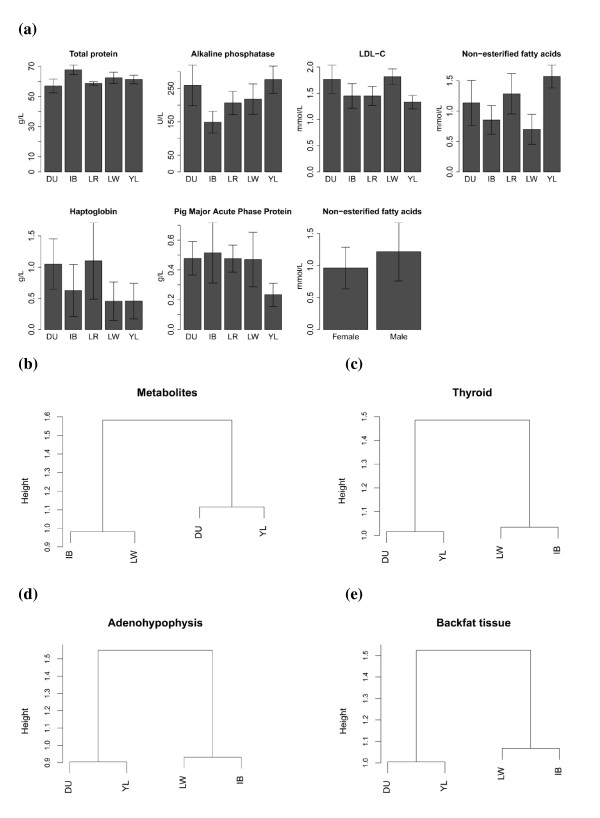
**Comparing the metabolic traits between breeds**. a) Bar plots of metabolic traits that significantly differed across sexes and breeds i.e. Duroc (DU), Iberian (IB), Landrace (LR), Large White (LW) and a Sino-European hybrid line (YL). b) Dendrogram of the four pig breeds (DU, IB, LR, LW) in terms of average standardized values for the 12 plasma metabolites. c-e) Dendrograms between breed z-scores for a subset of tissues i.e. thyroid (THYG), adenohypophysis (AHYP) and backfat (FATB).

The physiological relevance and main statistics of the 12 metabolites considered in this study are summarized in Table 1. Overall, sex had little influence. Given a p-value threshold of 0.05, only the NEFA levels differed between sexes, with male pigs having higher NEFA levels than female pigs (1.22 ± 0.46 mmol/L vs. 0.96 ± 0.33 mmol/L) (Figure 1). In comparison, breed was a greater source of variability. Breed effects were significant for six traits (P < 0.05). The most breed-biased trait was total protein content, followed by NEFA, ALP, LDL-C, haptoglobin and PigMAP. Sino-European hybrid pigs had the highest NEFA and ALP levels, but the lowest PigMAP and LDL-C levels, Iberian pigs had the highest total protein and PigMAP levels, but the lowest ALP level and a relatively low NEFA content and the Duroc and Large White pigs had the highest LDL-C levels (Figure 1). The correlation coefficients among the levels of the 12 metabolites are summarized in Additional file 1: Table S1. The strongest correlation was observed between LDL-C and cholesterol (r = 0.84), which is not unexpected since cholesterol is defined as the sum of LDL-C, HDL-C and other forms of lipoprotein associated cholesterol.

**Table 1:** Characteristics and statistics of the 12 plasma metabolites analyzed in this study

Metabolite	Physiological indications	Mean (SD)	P_sex_	P_breed_
**Glucose (mmol/L)**	diabetes, stress	4.05 (1.15)	0.50	0.09
**3-hydroxybutyrate (mmol/L)**	energy source of brain, rise when blood glucose is low	0.04 (0.02)	0.61	0.10
**NEFA (mmol/L)**	starvation, insulin resistance and blood pressure	1.08 (0.41)	0.025	0.0005
**Cholesterol (mmol/L)**	progression of atherosclerosis, diet	2.93 (0.37)	0.73	0.15
**HDL-C (mmol/L)**	inverse predictor of cardiovascular disease	1.07 (0.15)	0.08	0.16
**LDL-C (mmol/L)**	high level Associated with cardiovascular disease	1.57 (0.27)	0.67	0.002
**Triglyceride (mmol/L)**	atherosclerosis, heart disease and stroke, diet	0.77 (0.43)	0.83	0.43
**Total protein (g/L)**	reflects albumin concentration, infection, inflammation.	61.66 (4.88)	0.59	0.0003
**ALT (U/L)**	rises dramatically in acute liver damage	51.33 (7.75)	0.83	0.30
**ALP (U/L)**	rises with large bile duct obstruction, liver disease	219.0 (61.0)	0.98	0.0011
**Haptoglobin (g/L)**	infection, inflammatory and pathological lesion, stress	0.72 (0.48)	0.46	0.047
**PigMAP(g/L)**	infection, inflammatory and pathological lesion, stress	0.44 (0.17)	0.24	0.048

P_sex _and P_breed_: P value corresponding to significance of sex and breed effect by F test, respectively; non-esterified fatty acids (NEFA); alanine aminotransferase (ALT); alkaline phosphatase (ALP); pig major acute phase protein (PigMAP)

Differences in metabolite levels among breeds were also visualized with a dendrogram, these differences being defined as 1 - r, where r is the correlation coefficient between standardized average values of 12 metabolites in any two breeds. Note that a perfect positive correlation corresponds to 0, no correlation to 1 and a perfect negative correlation to 2 on the y axis (Figure 1b). To facilitate the comparison with the dendrograms built with gene expression data, only the 16 animals with transcriptome data were used. As shown in Figure 1b, the Iberian and Large White breeds were within the same clade, whereas the Duroc breed and the Sino-European hybrids clustered together in a distinct clade. The height of these two clades was approximately equal to 1, meaning that the metabolite levels between Iberian and Large White pigs, and between Duroc and Sino-European pigs were uncorrelated, whereas the total height of the tree was ~ 1.6, suggesting a negative correlation between clades. Notably, we observed similar patterns in dendrograms constructed using a Bayesian standardized measure of the breed's gene expression levels [7] in adenohypophysis, thyroid gland, backfat tissue, hypothalamus, and female gonad (Figure 1c-e).

### Association between transcriptome and plasma metabolites

Next, we investigated the association between metabolites and transcripts in each tissue separately across the 16 pigs (see methods above). A probeset was defined as a quantitative trait transcript (QTT) if its association with a particular metabolic trait achieved a nominal P value < 0.01. The number of QTT for the 12 metabolites in each tissue is shown in Table 2. For most of the metabolic traits, the number of QTT in the five tissues did not exceed the number expected by chance. Only three traits, ALT, HDL-C and NEFA measures had more than 500 QTT (FDR ~ 50%) detected in at least one tissue. For ALT, 3,322 QTT (FDR ~ 6%) were detected in the thyroid, which is much higher than the number of QTT associated with ALT in other tissues. For NEFA, we observed more than 500 QTT in four tissues: adenohypophysis, gonad, hypothalamus and thyroid. Note that fewer QTT were found in backfat tissue than in other tissues, although NEFA is mainly secreted by adipose tissue.

**Table 2:** Number of QTT for each plasma metabolite measured in five tissues

Metabolite	FATB	GONA	AHYP	THYG	HYPO
**Glucose**	108 (409)^1^	191 (234)	123 (215)	191 (185)	115 (180)
**3-hydroxybutyrate**	103 (159)	344 (259)	113 (258)	279 (148)	209 (290)
**Non-esterified fatty acids**	458 (201)	1113 (215)	1919 (209)	655 (214)	1003 (358)
**Cholesterol**	56 (197)	51 (201)	83 (259)	93 (338)	72 (365)
**HDL-C**	291 (157)	100 (173)	460 (205)	373 (191)	547 (541)
**LDL-C**	84 (262)	62 (323)	82 (374)	62 (283)	45 (656)
**Triglyceride**	185 (292)	117 (213)	273 (175)	304 (143)	318 (177)
**Total protein**	207 (311)	63 (192)	89 (146)	80 (162)	95 (176)
**Alanine aminotransferase**	166 (218)	613 (368)	112 (305)	3322 (193)	441 (232)
**Alkaline phosphatase**	138 (304)	175 (323)	202 (301)	96 (179)	57 (240)
**Haptoglobin**	84 (327)	45 (251)	70 (263)	118 (181)	72 (235)
**PigMAP**	51 (287)	254 (253)	100 (172)	99 (161)	58 (161)

^1^In brackets, number of QTT expected by random chance; backfat (FATB); gonad (GONA); adenohypophysis (AHYP); thyroid (THYG); hypothalamus (HYPO)

**Figure 2:**
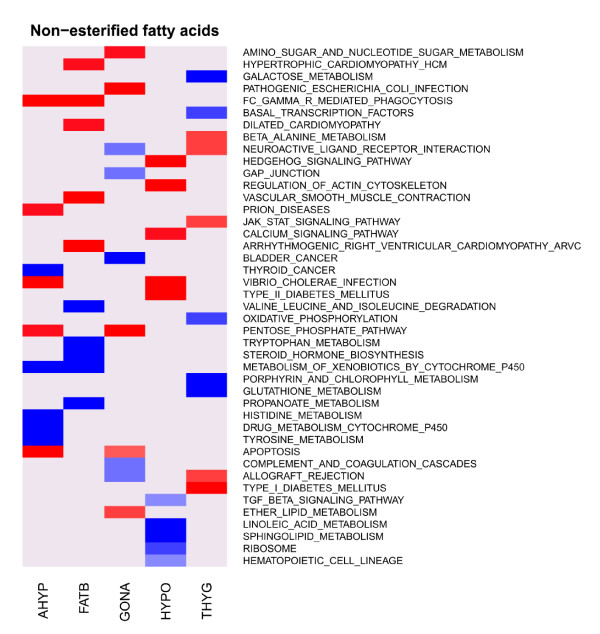
**Heat map of KEGG pathways enrichment scores for non-esterified fatty acids in five tissues**. Red (blue) denotes top five pathways with positive (negative) normalized enrichment scores in gene set enrichment analysis (GSEA) for backfat (FATB), gonad (GONA), adenohypophysis (AHYP), thyroid (THYG), hypothalamus (HYPO).

To assess the tissue specificity of associations between transcripts and metabolites and to which extent QTT and functional gene sets associated with a particular metabolite were shared across tissues, we used two approaches: QTT overlap analysis and GSEA. To evaluate the overlap of QTT, we examined whether the number of QTT shared by any two tissues was significantly larger than random expectations using Fisher's exact test. Generally, a very limited overlap of QTT across tissues was observed for most of the traits. Excessive QTT overlaps between tissues (P value < 10^-4^) were observed only for HDL-C and NEFA levels (Table 3). The QTT enriched for genes involved in a biological process i.e. RNA processing (Table 3) were those shared by hypothalamus and thyroid and associated with HDL-C. GSEA associates gene sets, rather individual genes, to a given trait, and has been shown to have greater power in finding similarities between two independent studies than in a single-gene analysis [10]. Figure 2 shows the top 10 KEGG pathways with the most significant normalized enrichment scores, five positive (red) and five negative (blue) for NEFA in the five tissues. Similar to the QTT overlaps, a limited number of pathways were preserved across tissues. A similar situation was observed for other metabolic traits. Overall, these observations suggest that the associations between transcriptome and metabolites are highly tissue-specific. This is also in agreement with our previous analyses [7,15], that highlighted that the factor with the largest effect on transcriptome was tissue.

**Table 3:** Tissue pairs with a significant number of overlapping QTT

Metabolite	Tissue pairs	Count (fold)	Bonferroni P value	GO terms
**HDL-C**	FATB-THYG	17 (3.8)	0.000396	-
**HDL-C**	AHYP-THYG	25 (3.5)	7.92E-06	-
**HDL-C**	AHYP-HYPO	40 (3.8)	5.18E-11	-
**HDL-C**	THYG-HYPO	50 (5.9)	3.99E-22	RNA processing
**NEFA**	GONA-AHYP	211 (2.4)	1.22E-31	-

Non-esterified fatty acids (NEFA); backfat (FATB); gonad (GONA); adenohypophysis (AHYP); thyroid (THYG); hypothalamus (HYPO)

### Gene co-expression networks

A gene co-expression network is a representation of how transcripts are correlated. Genes within the same biological pathway can be highly correlated and therefore grouped into the same module. Using weighted gene co-expression network analysis, the QTT for each of the 12 metabolic traits in each of the five tissues were clustered into one to four modules. Because the networks were constructed using probesets separately for each tissue, we refer to these networks as single-tissue networks. Furthermore, we examined the biological significance of these modules by gene ontology (GO) categories (including biological processes, molecular function and cellular component) and KEGG pathways enrichment analysis. The enrichment of these gene categories was assessed by p values corrected by the Benjamini and Hochberg approach [16].

Five of the 12 traits, i.e. NEFA, ALT, HDL-C, glucose and triglyceride levels were found to have a least one module enriched for genes in certain KEGG or GO categories (P_Benjamini _< 0.05, Table 4 and Additional file 2: Table S2). The most striking result was found for NEFA, for which enrichment of functional categories was observed in four tissues. The backfat module was enriched in oxidation reduction and biosynthesis of unsaturated fatty acids. The gonad module was enriched in genes participating in the regulation of protein and nucleotide metabolisms, in cell-cell signaling and T cell proliferation. We observed that both adenohypophysis (30 genes) and hypothalamus (44 genes) modules were enriched for genes involved in protein transport, however, only three genes (*IPO9, PACS1 *and *PSEN1*) were shared between tissues. This is consistent with the highly tissue-specific pattern of associations mentioned above. For ALT, the most remarkable tissue is thyroid, for which the 3322 QTT were grouped into a single module, 96% of the QTT being positively associated with ALT. This module is enriched in genes related to a large variety of functional categories (Table 4). The gonad module was enriched for genes involved in cell adhesion, leukocyte trans-endothelial migration, nucleoside triphosphate metabolism and blood vessel development.

**Table 4:** Enrichment of gene categories in different tissue modules for NEFA, HDL-C, triglyceride, glucose and ALT levels

Metabolite	FATB	GONA	AHYP	THYG	HYPO
**Glucose**		RNA binding and splicing			

**NEFA**	oxidation reduction biosynthesis of unsaturated fatty acid coenzyme binding mitochondrion	regulations of protein, nucleotide metabolism cell-cell signaling T cell proliferation synaptic transmission muscle and skeletal development behavior	protein transport and localization calcium ion binding neuron projection presynaptic membrane contractile fiber dendritic shaft		protein transport learning and memory proton transporting ATPase complex synapse; dendritic shaft cell junction

**HDL-C**	lipoprotein particle			RNA processing; ribosome	RNA splicing

**Triglyceride**				Alzheimer's disease monosaccharide catabolic process	

**ALT**		tight junction cell adhesion leukocyte transendothelial migration nucleoside triphosphate metabolic process blood vessel development regulation of cell motion polysaccharide and heparin binding		ECM receptor interaction focal adhesion cell motion neuron differentiation cell-cell signaling muscle, heart and bone development regulation of transcription and metabolic processes response to wounding learning and memory	

Non-esterified fatty acids (NEFA); alanine aminotransferase (ALT); backfat (FATB);, gonad (GONA); adenohypophysis (AHYP); thyroid (THYG); hypothalamus (HYPO)

**Figure 3:**
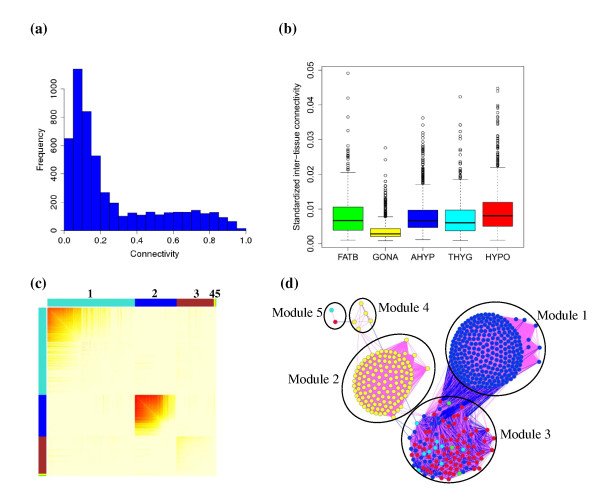
**Analysis of multiple tissue network for non-esterified fatty acids**. a) Distribution of probeset connectivity in the multiple-tissue network. b) Box plot of standardized inter-tissue connectivity of genes in the five tissues i.e. backfat (FATB), gonad (GONA), adenohypophysis (AHYP), thyroid (THYG) and hypothalamus (HYPO). c) Heat map for the multiple-tissue network, color shades i.e., from white to red represent the correlation strength between a pair of probesets; different modules are indicated by different colors in the row and column box, and ordered by size (the module labels are shown on top of the graph); the genes within modules in the rows and columns are sorted according to their intramodular connectivity. d) A subset of the multiple-tissue network containing nodes that are QTT for NEFA in the five tissues; here, the nodes represent the top 10% probesets with the highest intramodular connectivity in each of the four modules; node colors denote the tissues: red (hypothalamus), blue (adenohypophysis), yellow (gonad), cyan (thyroid) and green (backfat); two nodes were connected with an edge if their correlation was significant (nominal P < 10^-4^, FDR < 0.05), the pink (blue) edge indicates a positive (negative) correlation.

The previous results were obtained from analyses on separate tissues. Because endocrine tissues regulate the homeostasis of plasma metabolites through the secretion of hormones collaboratively rather than independently, a deeper understanding of the biology should be gained by considering several tissues simultaneously. We assumed that inter-tissue communications would be reflected in the inter-tissue gene correlations. To investigate the inter-tissue connections at the gene expression level, we constructed a multiple-tissue gene co-expression network that contained 5148 nodes (QTT) associated with NEFA from the five tissues. We focused on NEFA because it was the metabolite for which the largest number of QTT and biologically meaningful modules across the five tissues was found (Tables 2 and 4). In this multiple-tissue network, a large proportion of the nodes were loosely connected, whereas a small proportion of nodes were highly connected (Figure 3a). The hypothalamus genes had the highest average inter-tissue connectivity, while the gonad genes had the lowest (Figure 3b). We also assessed the connection strength between tissues. Interestingly, the strongest connection was observed between hypothalamus and adenohypophysis (Additional file 3: Table S3), two tissues that are closely related. The entire network was divided into five modules (Figure 3c). Module 1 was enriched for adenohypophysis probesets, modules 2 and 4 were enriched for gonad probesets, whereas module 3 was overrepresented with hypothalamus and thyroid probesets. Module 5 was not enriched for any tissue (Additional file 4: Table S4).

Highly connected (hub) nodes constitute the backbones of a network structure. In Figure 3d, we show a subset of the entire network using the top 10% probesets with the highest intra-modular connectivity (hub nodes). Several interesting observations can be made. All hub nodes in module 1 corresponded to adenohypophysis, while all hub nodes in modules 2 and 4 corresponded to gonad, these modules possibly reflecting biological processes that operate within tissues. In contrast, hub nodes in module 3 corresponded to four tissues including hypothalamus, thyroid, adenohypophysis and backfat, suggesting that the genes in this module could be part of gene regulation pathways that are involved in communications between tissues. Notice that 64% (73/114) of the hub genes in module 3 corresponded to hypothalamus, which is regarded as an organ integrating information from the body's nutritional and hormonal signals. Both positive and negative correlations among hub nodes were present in module 3, indicating the existence of feedback signaling. In comparison, only positive correlations among probesets within the three other modules were observed. There are many more links between module 1 and module 3 than between any other pair of modules. Many of these are links between hypothalamus and adenohypophysis genes. Interestingly, hormone secretion in the adenohypophysis is directly regulated by neurons in the hypothalamus. Thus, these observations emphasize the central role of the hypothalamus with regard to gene regulation networks.

## Discussion

Plasma metabolite levels are main indicators of endocrine status, including health status, and are potential predictors of performance. In this study, a survey of 12 plasma metabolites showed that six metabolites, including total protein, NEFA, ALP, LDL-C, haptoglobin and PigMAP are affected by breed (*P *< 0.05) and therefore have a partial genetic cause. The Iberian pig, which is fatter and grows more slowly than commercial pig breeds, has the highest average levels of total protein and PigMAP, but the lowest level of ALP. Interestingly, ALP is reported to be associated with body weight in pigs [1]. The Sino-European hybrid pigs have lower haptoglobin and PigMAP average levels which are positively associated with inflammatory processes. This suggests that the Sino-European hybrid pigs could have a weaker inflammatory response as compared to e.g., Duroc and Landrace breeds (Figure 1a). Notably, we observed a similar pattern of correlation among breeds in terms of both the levels of the 12 metabolites and the transcriptome in multiple tissues (Figure 1b-e).

The endocrine glands play important roles in maintaining homeostasis of metabolites in blood. Here, we report an association analysis between gene expression profiles in five endocrine tissues and plasma metabolites in pigs. The associations were found to be highly tissue-specific, as suggested by the limited overlap of QTT and biological pathways in the five tissues for all the metabolites. The QTT for NEFA, ALT, HDL-C, triglyceride and glucose within each tissue were grouped into biologically meaningful sub-networks. Furthermore, we constructed a multiple-tissue network using QTT from the five tissues for NEFA.

Overall, the FDR of the associations between probesets and metabolites was high at the current significance threshold (P < 0.01) and a similar high FDR was also observed at a stricter threshold (P < 0.001). This is likely due to the limited size of the sample (N = 16). Yet, we did find a significant increase in the number of QTT for NEFA and ALT, and the QTT within tissues were grouped into biologically meaningful modules (detailed below).

The limited overlap between QTT and gene pathways across tissues suggests that the associations between endocrine transcriptome and biochemical traits were highly tissue-specific. This is in agreement with our previous analyses of the data as well [15] and with the literature in general. For instance, Yang et al. [17] have reported a minimal overlap and very different functional categories of sexually dimorphic genes in brain, liver, adipose and muscle of mice. Therefore, candidate genes or gene pathways e.g., obtained from genome-wide association studies should be investigated in the context of specific tissues.

### Single tissue network

The most significant observations regarding QTT number concerned NEFA. NEFA derive from the hydrolysis of triglycerides in adipose tissue or lipoproteins, circulate in the blood and serve as source of energy (especially for heart and muscle) and cellular signaling messengers. In the backfat module, we found that genes involved in the biosynthesis of unsaturated fatty acids (such as *ELOVL6, ACOT4, ACOT7*, *HSD17B12, PECR *and *SCD*) were negatively correlated with NEFA, suggesting that the synthesis of unsaturated fatty acids was repressed in animals with higher plasma NEFA levels. Moreover, other genes involved in fatty acid and lipid metabolisms (such as *DECR1, ACADL, ACOX2, DCI, ECHDC2, FABP3, FASN, LIPA, PRDX6*, *ENPP2, DDHD1, DGAT2 *and *SCP2*) were also found negatively correlated with NEFA in this module. The hypothalamus module for NEFA was enriched for genes related to synapses, learning and memory. Many genes participating in protein transport and localization processes like *SENP1, CDK5, SYNGR1, SNAP23, RIMS1 *and *YWHAZ *are also active at synapses. Synaptic plasticity in the hypothalamus is known to be associated with nutritional state [18]. In the adenohypophysis module, genes involved in calcium ion binding, protein transport and localization, neuron projection and in the presynaptic membrane were overrepresented. The importance of calcium-dependent electrical activity in adenohypophysis cells has been reviewed, e.g., by [19]. Both in vitro [20] and in vivo [21] experiments have shown that changing NEFA concentrations can alter pituitary hormone secretion in pigs. Both in humans and dog, it was shown that the plasma NEFA level increases after administration of growth hormone [22], NEFA in turn can block growth hormone secretion [23]. Thus, in general, we observe that enriched functional categories often have a physiological interpretation.

For ALT, the most relevant tissues in this analysis are the thyroid and gonad (Tables 2 and 4). The observed large number (3322) of QTT and wide range of functional categories in thyroid suggest a close relationship between thyroid function and plasma ALT levels. It is well known that ALT blood levels reflect the liver condition since clinical links between the thyroid and liver are well documented. Liver metabolizes the thyroid hormone, which in turn influences the liver function and thyroid disorders are often associated with an elevation of ATL [24]. In the gonad module, we checked the genes in the enriched functional categories using DAVID online tools http://david.abcc.ncifcrf.gov/, and found that many genes (*CLDN3, CLDN4, PTK2B*, *EPAS, CDH1, CDH2, TYMP, TGFA, WT1, CTGF, FN1 *and *ITGB3*) related to cell adhesion or migration were associated to ovarian tumors. Moorthy et al. (2005) reported that administration of gonadal hormones like estradiol and progesterone decreased ALT levels in heart, liver, kidney and uterus in naturally menopausal rats [25].

For HDL-C, the backfat module, was slightly enriched for apolipoprotein genes including *APOB*, *APOA4*, *APOC3*, *APOC4 *and *APOH *(P_Benjamini _= 0.061). This observation is unexpected, since no evidence was found to support the synthesis of these apolipoproteins in adipose tissue. Both thyroid gland and hypothalamus modules contain a group of genes participating in mRNA processing specifically mRNA splicing. Alternative pre-mRNA splicing plays an important role in the control of neuronal development and function [26]. Thyroid hormones and their receptors have been shown to stimulate reverse cholesterol transport in animal models [27].

### Multiple-tissue network

To explore the connections between tissues at the gene expression level, we built a co-expression network containing all the QTT for NEFA from the five tissues (Figure 3). Module 3, in which hypothalamus genes are overrepresented, appears to be particularly interesting. The top 10% most connected genes in this module are from four different tissues and might constitute core regulation pathways involved in communication between tissues. Additionally, we have also shown that genes have a significantly higher average inter-tissue connectivity in the hypothalamus than in other tissues (Figure 3b). These observations emphasize the central role of hypothalamus genes in the multiple-tissue co-expression network. Dobrin et al. (2009) constructed inter-tissue co-expression networks between hypothalamus, liver and adipose tissue. Their results also suggested the hypothalamus as the controlling tissue since asymmetric connectivity was more common in the hypothalamus than in other tissues. e.g., the most connected hypothalamus gene, *Aqp5 *was linked to 169 adipose genes, while adipose gene *Aqp5 *was only linked to two hypothalamus genes. Interestingly, the hypothalamus is known as an organ that integrates and responds to signals from peripheral tissues [28,29].

More links were found in hub genes between modules 1 and 3 than between any other modules (Figure 3d), suggesting that the genes in these two modules act in a more coordinate fashion. Several hypothalamus genes (*FAM69B, NPTXR, RUNDC3A*, *N4BP2L2, KIAA1429, SNURF *and *KCTD20*) and a backfat gene (*RUNDC3B*) in module 3 were highly connected to hub genes in module 1. Furthermore, we examined the hub genes in module 3 (Additional file 5: Table S5) using DAVID online tools [14], and highlighted the genes associated with functions in corresponding tissues. We found genes in the hypothalamus that were related to the differentiation and development of the central nervous system (*ATP7A, CDK5*, *HPRT1 *and *SS18L1*) and to protein transport and localization (*ARFIP1, RAB6B, SENP2, C11orf2, PACS1, RIMS1 *and *TNKS*). Most of these genes are relevant to neuron function or energy balance e.g., *CDK5 *is a member of the cyclin dependent kinase family, and serves as an essential modulator of synaptic function and plasticity [30]. *RAB6B *is a GTPase predominantly expressed in brain that has been suggested to participate in retrograde transport of cargo in neuronal cells [31]. This gene was also up-regulated in the brain of mice fed with omega 3 polyunsaturated fatty acid enriched diet [32]. *TNKS *is a Golgi associated poly-ADP-ribose polymerase gene, abundantly expressed in brain. *TNKS*-deficient mice show an increase in energy expenditure, fatty acid oxidation and insulin simulated glucose utilization [33]. Two transcripts in the backfat module correspond to *UGP2*, which is involved in the synthesis of UDP-glucose, the precursor of glycogen in liver and muscle tissue, and of lactose in lactating mammary gland. Among the adenohypophysis genes, *FTO, RHOB *and *ELP2 *are related to the function of the adenohypophysis. *FTO *is a well studied gene that is abundantly expressed in the hypothalamus and adenohypophysis and related to food intake and obesity [34,35]. *RHOB *is a GTP-binding protein involved in vesicular trafficking in anterior pituitary cells [36]. *ELP2 *is an isoform of steroidogenic factor 1 (SF1), and plays an important role on pituitary gonadotrope function.

## Conclusions

Our results suggest a partial hereditary basis for some metabolite levels, like total protein, NEFA, ALP, LDL-C, haptoglobin and PigMAP. Single-tissue gene co-expression networks were composed of highly connected modules associated with metabolites (especially NEFA) that had a biologically meaningful role. These networks were, in general, tissue-specific. Finally, the multiple-tissue network emphasized the central role played by the hypothalamus.

## Competing interests

The authors declare that they have no competing interests.

## Authors' contributions

MPE and BY designed the study, AB and YS provided the metabolite measurements, BY analyzed data. BY and MPE wrote the manuscript with help from the rest of authors. All authors read and approved the final manuscript.

## Supplementary Material

Additional file 1 **Pearson correlation coefficients for pairs of traits**. This file provides the analysis of pair-wise Pearson correlation coefficients for levels of the 12 metabolites. Click here for file

Additional File 2 **Enrichment of KEGG or GO categories in the modules of the five tissues for five plasma metabolites**. This file provides detailed KEGG and GO categories that are significantly (P_Benjamini _< 0.05) enriched in QTT modules associated with nonesterified fatty acids, HDL-C, Triglyceride, Glucose and Alanine aminotransferase. Click here for file

Additional File 3 **Strengths of connection between any two tissues in terms of inter-tissue correlations of gene expression traits**. This file provides a table of connection between any tissue pairs in terms of inter-tissue correlations of gene expression traits. Click here for file

Additional File 4 **Enrichment of tissue probesets in modules of multiple-tissue network for NEFA**. This file provides results of enrichment of probesets from a certain tissue in the modules of multiple-tissue network that associated with non-esterified fatty acids. Click here for file

Additional File 5 **Top 10% probesets in module 3 of the multiple-tissue network for NEFA**. This file provides origin of tissue, gene symbols, Entrez gene ID, Intramodular connectivity and annotations for top 10% probesets in module 3 of the multiple-tissue network that associated with non-esterified fatty acids. Click here for file
